# Beta Blockers Improve Prognosis When Used Early in Patients with Cardiogenic Shock: An Analysis of the FRENSHOCK Multicenter Prospective Registry

**DOI:** 10.3390/ph16121740

**Published:** 2023-12-18

**Authors:** Laura Sofia Cardelli, Miloud Cherbi, Fabien Huet, Guillaume Schurtz, Eric Bonnefoy-Cudraz, Edouard Gerbaud, Laurent Bonello, Guillaume Leurent, Etienne Puymirat, Gianni Casella, Clément Delmas, François Roubille

**Affiliations:** 1 Cardiology Department, Ospedale Versilia, 55049 Camaiore, Italy; cardellilaura.med@gmail.com; 2 Intensive Cardiac Care Unit, Rangueil University Hospital, 31059 Toulouse, Francedelmas.clement@chu-toulouse.fr (C.D.); 3 Department of Cardiology, Centre Hospitalier Bretagne Atlantique, 56000 Vannes, France; 4 Department of Cardiology, Urgences et Soins Intensifs de Cardiologie, CHU Lille, University of Lille, Inserm U1167, 59000 Lille, France; 5 Intensive Cardiac Care Unit, Lyon Brom University Hospital, 69677 Lyon, France; 6 Intensive Cardiac Care Unit and Interventional Cardiology, Hôpital Cardiologique du Haut Lévêque, 5 Avenue de Magellan, 33604 Pessac, France; edouard.gerbaud@chu-bordeaux.fr; 7 Intensive Care Unit, Department of Cardiology, Assistance Publique-Hôpitaux de Marseille, Hôpital Nord, Aix-Marseille University, 13015 Marseille, France; 8 Department of Cardiology, CHU Rennes, Inserm, LTSI—UMR 1099, Univ Rennes 1, 35000 Rennes, France; 9 Assistance Publique-Hôpitaux de Paris (AP-HP), Hôpital Européen Georges Pompidou, 75015 Paris, France; 10 Cardiology Department, Ospedale Maggiore, 40131 Bologna, Italy; 11 REICATRA, Institut Saint Jacques, 31059 Toulouse, France; 12 PhyMedExp, INSERM, CNRS, Cardiology Department, INI-CRT, Université de Montpellier, 34295 Montpellier, France

**Keywords:** betablocker, cardiogenic shock, acute heart failure, one-month mortality, one-year mortality

## Abstract

Background: Beta blockers (BBs) are a cornerstone for patients with heart failure (HF) and ventricular dysfunction. However, their use in patients recovering from a cardiogenic shock (CS) remains a bone of contention, especially regarding whether and when to reintroduce this class of drugs. Methods: FRENSHOCK is a prospective multicenter registry including 772 CS patients from 49 centers. Our aim was to compare outcomes (1-month and 1-year all-cause mortality) between CS patients taking and those not taking BBs in three scenarios: (1) at 24 h after CS; (2) patients who did or did not discontinue BBs within 24 h; and (3) patients who did or did not undergo the early introduction of BBs. Results: Among the 693 CS included, at 24 h after the CS event, 95 patients (13.7%) were taking BB, while 598 (86.3%) were not. Between the groups, there were no differences in terms of major comorbidities or initial CS triggers. Patients receiving BBs at 24 h presented a trend toward reduced all-cause mortality both at 1 month (aHR = 0.61, 95% CI 0.34 to 1.1, *p* = 0.10) and 1 year, which was, in both cases, not significant. Compared with patients who discontinued BBs at 24 h, patients who did not discontinue BBs showed lower 1-month mortality (aHR = 0.43, 95% CI 0.2 to 0.92, *p* = 0.03) and a trend to lower 1-year mortality. No reduction in outcomes was observed in patients who underwent an early introduction of BB therapy. Conclusions: BBs are drugs of first choice in patients with HF and should also be considered early in patients with CS. In contrast, the discontinuation of BB therapy resulted in increased 1-month all-cause mortality and a trend toward increased 1-year all-cause mortality.

## 1. Introduction

Betablockers (BBs) are one of the first-line drug classes recommended to improve morbidity and mortality in patients with chronic heart failure (HF), with reduced left ventricular ejection fraction (LVEF).

Data are mainly derived from studies performed in stable outpatients. Recently, the STRONG HF trial showed that an early introduction of full doses of guideline-directed drug treatment (BBs, renin-angiotensin blockers, and mineralocorticoid receptor antagonists) was feasible for patients admitted for acute HF (AHF), after stabilization [1]. However, no robust data are currently available to guide physicians on whether to keep/reintroduce these therapies in the cardiac care unit (CCU), especially after resolved cardiogenic shock (CS), or even what could be the ideal timing for reintroduction [1,2,3]. Data on BB introduction following CS are very sparse and derived predominantly from the ischemic cardiomyopathy population, with no data regarding CS triggered by other etiologies [4,5]. BBs remain contraindicated in states of hemodynamic instability at risk of cardiogenic shock because of their negative inotropic and chronotropic effects [6,7]. Thus, most physicians are still reluctant to initiate early therapy in the most severe patients, mainly because of the risk of hemodynamic destabilization and its potential negative impact on patient outcomes. However, the early initiation of in-hospital BB therapy is generally well tolerated and results in a high rate of use after discharge at the recommended doses [8,9]. Current guidelines are elusive regarding the best way to start BB therapy in patients hospitalized for AHF. They recommend an early (as soon as possible) introduction. That means with great caution and only when the patient is considered stabilized [6]. However, it remains unclear when and how physicians should start administering BBs, resulting in great heterogeneity in the management of patients with more severe AHF [10]. The current consensus is to continue treatment with BBs in patients who were previously taking them in the absence of hemodynamic instability, since it has been shown that dose reduction or discontinuation of BBs during AHF episodes may result in unfavorable outcomes [11].

Based on a large, unselected CS registry from all etiologies, we aimed to compare patients taking or not taking BBs and continuing or not continuing BBs 24 h after CS onset regarding baseline characteristics, associated management, and 1-month and 1-year outcomes.

## 2. Results

### 2.1. Baseline Characteristics and CS Management According to the Intake of Betablockers at 24 h

**Figure 1:**
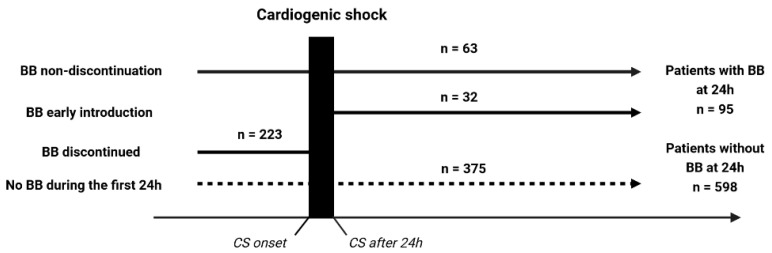
Study population. BB, betablocker, CS, cardiogenic shock.

Of 772 patients, 79 patients were excluded from the current analysis due to missing data. Thus, 693 patients were enrolled: 95 in the BB group and 598 in the non-BB group (Figure 1). Their clinical characteristics are shown in Table 1. The mean age was 66 ± 14.6 years, with a predominance of men (71.4%).

The two groups did not differ in terms of the history of cardiomyopathy, the proportion of patients with an implantable cardioverter–defibrillator (ICD), and previous NYHA functional status. The BB group had more hypertension (60% vs. 45.5%, *p* = 0.01) and diabetes (37.9% vs. 26.6%, *p* = 0.03), while there was a higher prevalence of males in the non-BB group (73.9% vs. 55.8%, *p* < 0.01). However, patients in the BB group were taking more anticoagulants (both vitamin K antagonists and direct oral anticoagulants) (41.1% vs. 27.1%, *p* < 0.01) and loop diuretics (58.9% vs. 47.5%, *p* = 0.046).

There were no differences between the two groups in terms of major comorbidities nor differences for CS triggers (Table 1). Notably, there were no differences between the two groups in terms of supraventricular tachycardias (13.7% vs. 13.9%, *p* = 1.00) and ventricular arrhythmias (15.8% vs. 11.9%, *p* = 0.31) as triggers of CS.

Patients taking BB at 24 h had better LVEF (29.9 ± 13 vs. 26.1 ± 13, *p* < 0.01), lower lactates (2.2 (1.9–3.0) vs. 3 (2–5) mmol/L, *p* < 0.01), and better renal function (*p* = 0.01) but also higher SBP (108.2 ± 21 vs. 101.1 ± 25.6 mmHg, *p* < 0.01) at admission (Table 1).

In addition, BB patients were less frequently treated with inotropes/vasopressors (62% vs. 85% and 26.3% vs. 56.3%, respectively; *p* < 0.01 for both), invasive ventilation (15.8% vs. 39.2%, *p* < 0.01), and aMCS (9.5% vs. 18.8%, *p* = 0.03) (Table 2).

**Figure 2:**
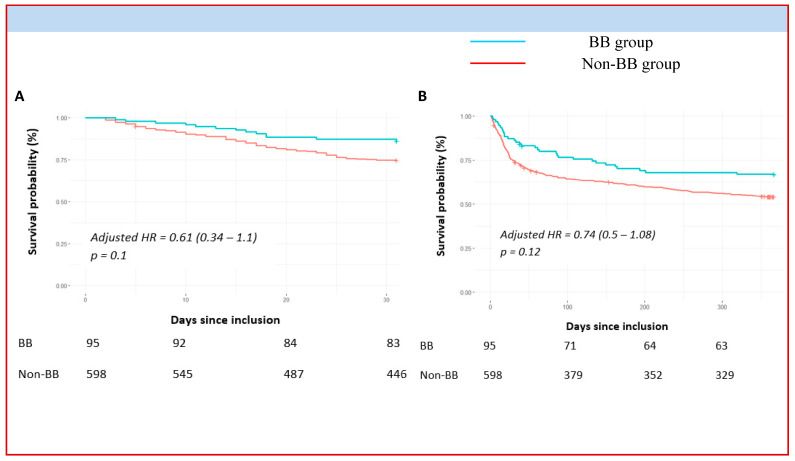
Short- and long-term mortality outcomes after CS according to treatment with betablockers at 24 h (BB vs. non-BB). BB = betablocker, HR = hazard ratio, Panel (**A**) represents 1-month overall mortality. Panel (**B**) focus on 1-year mortality. The cumulative incidences of 1-year and 1-month mortality were estimated with the use of the Kaplan–Meier method; hazard ratios and 95% confidence intervals were estimated with the use of Cox regression models. According to significant characteristics found as independent predictive factors in multivariable analyses, 1-year mortality was adjusted for age, current smoking, active cancer, use of norepinephrine, acute renal replacement therapy, use of aMCS, and non-observance triggers. We adjusted 1-month mortality for age, diabetes mellitus, current smoking, previous ICD, use of diuretics, acute renal replacement therapy, use of aMCS, and LVEF ≤ 30%.

A trend of reduction in all-cause mortality, although not significant, was observed at 1 month (aHR = 0.61, 95% CI 0.34 to 1.1, *p* = 0.10) and at 1 year (aHR = 0.74, 95% CI 0.5 to 1.08, *p* = 0.12) for the BB group after adjustment for clinical and therapeutic severity factors of CS (Figure 2).

### 2.2. Betablockers Non-Discontinuation at 24 h

At admission, 286 patients were on BBs. A total of 223 discontinued BBs during the first 24 h, while 63 did not. The comparison of initial characteristics and CS triggers (shown in Supplementary Tables S1 and S2) showed no substantial differences between these two subgroups, except a greater proportion of males among patients who discontinued BBs (71.3% vs. 44.4%, *p* < 0.01). Notably, also in this case, there were no differences between the two groups in terms of supraventricular tachycardias (14.3% vs. 17.5%, *p* = 0.7) or ventricular arrhythmias (14.3% vs. 11.2%, *p* = 0.51) as CS triggers.

Moreover, patients who discontinued BBs during the first 24 h presented lower LVEF (26.3 ± 13.4 vs. 30.2 ± 13.4, *p* = 0.03), poorer renal function (*p* = 0.02), and lower SBP at admission (99.1 ± 26 vs. 106.7 ± 20.7 mmHg, *p* < 0.01). This group of patients was also more often treated with inotropes/vasopressors (85.1% vs. 58.7% and 51.4% vs. 22.2%, respectively; *p* < 0.01 for both) and invasive ventilation (31.5% vs. 12.7%, *p* < 0.01).

**Figure 3:**
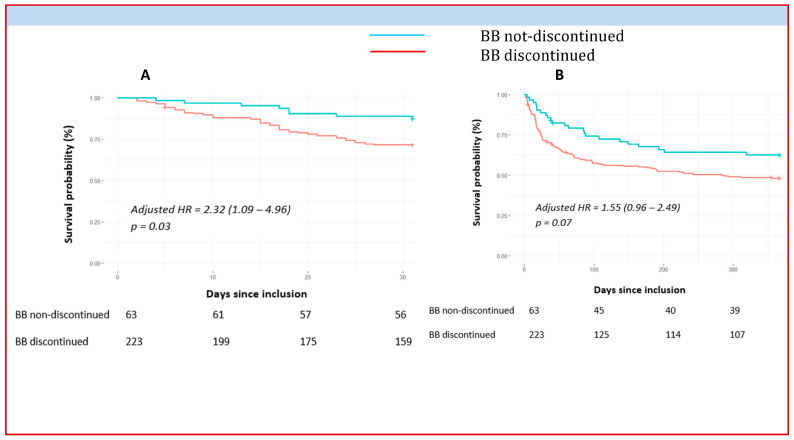
Short- and long-term mortality outcomes after CS according to discontinuation of betablockers within the 24 first hours. BB = betablocker, HR = hazard ratio, Panel (**A**) represents 1-month overall mortality. Panel (**B**) focuses on 1-year mortality. The cumulative incidences of 1-year and 1-month mortality were estimated with the use of the Kaplan–Meier method; hazard ratios and 95% confidence intervals were estimated with the use of Cox regression models. According to significant characteristics found as independent predictive factors in multivariable analyses, 1-year mortality was adjusted for age, current smoking, active cancer, use of norepinephrine, acute renal replacement therapy, use of aMCS, and non-observance triggers. We adjusted 1-month mortality was adjusted for age, diabetes mellitus, current smoking, previous ICD, use of diuretics, acute renal replacement therapy, use of aMCS, and LVEF ≤ 30%.

Patients who discontinued BBs at 24 h showed higher 1-month all-cause mortality (aHR = 2.32, 95% CI (1.09–4.96), *p* = 0.03) than those who continued with BBs (Figure 3), even after adjustment for CS severity factors. In addition, there was a trend toward an increase in 1-year all-cause mortality in patients discontinuing BBs (aHR = 1.55, 95% CI 0.96–2.49, *p* = 0.07).

### 2.3. Early Betablocker Introduction (<24 h)

Among the 407 patients without BBs at admission, the early introduction (≤24 h) of BB therapy involved 32 patients.

The two subgroups (early versus delayed BBs introduction subgroup) had similar CS triggers and clinical and management characteristics (Supplementary Tables S3 and S4). However, the patients in the early BB introduction subgroup were more likely to have diabetes (40.6 vs. 21.7%, *p* = 0.03). Notably, there were no differences between the two groups in terms of supraventricular tachycardias (12.5% vs. 11.7%, *p* = 0.78) and ventricular arrhythmias (18.8% vs. 12.3%, *p* = 0.28) as CS triggers.

Patients with early BB introduction showed lower blood lactate values (2 (1.7–2.9) vs. 3 (2–5) mmol/L, *p* = 0.047) and higher SBP values (111.2 ± 21.2 vs. 102.2 ± 25.3 mmHg, *p* < 0.01) at admission. In contrast, patients in the non-early BB introduction subgroup were more often treated with inotropes/vasopressors (84.8% vs. 31.3%, *p* = 0.03; 59.2% vs. 34.4%, *p* < 0.01, respectively) and with invasive ventilation (43.7% vs. 21.9%, *p* = 0.02). No differences were found between the groups in terms of aMCS use (18.8% vs. 20.3%, *p* = 1.00).

**Figure 4:**
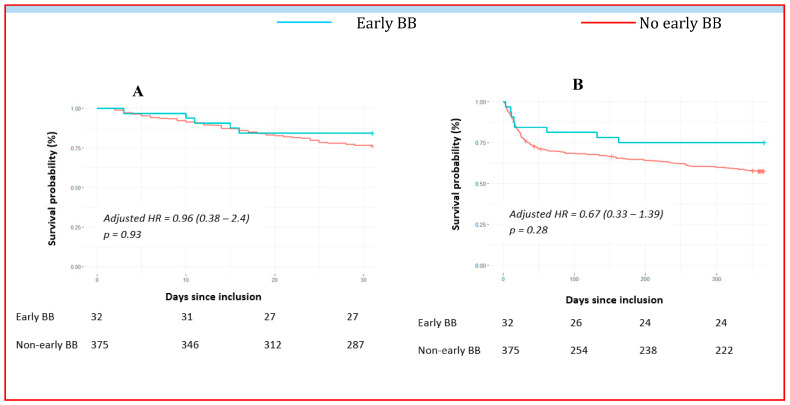
Short- and long-term mortality outcomes after CS according to early introduction of betablockers (early BB vs. non-early BB). BB = betablocker, HR = hazard ratio, Panel (**A**) represents 1-month overall mortality. Panel (**B**) focuses on 1-year mortality. The cumulative incidences of 1-year and 1-month mortality were estimated with the use of the Kaplan–Meier method; hazard ratios and 95% confidence intervals were estimated with the use of Cox regression models. According to significant characteristics found as independent predictive factors in multivariable analyses, 1-year mortality was adjusted for age, current smoking, active cancer, use of norepinephrine, acute renal replacement therapy, use of aMCS, and non-observance triggers. We adjusted 1-month mortality for age, diabetes mellitus, current smoking, previous ICD, use of diuretics, acute renal replacement therapy, use of aMCS, and LVEF ≤ 30%.

After adjusting for clinical and therapeutic severity factors for CS, early BB introduction resulted in no difference in 1-month (aHR = 0.96, 95% CI 0.38–2.4, *p* = 0.93) or 1-year all-cause mortality (aHR = 0.67, 95% CI 0.33–1.39, *p* = 0.28) between group (Figure 4).

## 3. Discussion

Based on the largest European prospective, observational, multicenter registry on CS from a wide spectrum of etiologies, we showed several points: (1) the use of BBs within 24 h of CS did not increase 1-month and 1-year all-cause mortality; (2) BB continuation in the early period of CS was not associated with increased short- and long-term mortality, but rather, BB discontinuation was associated with increased 1-month all-cause mortality; (3) early (≤24 h) BB introduction did not significantly improve prognosis but still had a positive trend; and (4) patients treated with BBs within 24 h of CS generally had less severe prior cardiomyopathy and less severe forms of CS.

Despite significant medical advances in recent years, the mortality rate of CS remains high, standing at 49.4% at 30 days, 60.4% at 1 year, and 62.5% in the long term (≥2 years) [12].

The benefits of BBs in chronic HF with reduced ejection fraction are well established [6], and there is a strong consensus to continue BB treatment while patients undergo AHF to improve survival [7,13,14,15,16,17,18]. Dose reduction or discontinuation of BBs in this setting has been shown to cause poor outcomes, and this should be limited to patients with refractory marked congestion, hemodynamic instability, major right ventricular failure, or severe renal impairment [11,19].

Over the years, evidence has also accumulated for patients with CS. However, to date, no solid data are available to guide physicians in initiating these therapies in patients with resolved CS, especially in ICU/CCU [6]. The early initiation of BB therapy in patients with AMI and risk factors for CS does not appear to be associated with an increased risk of shock [5]. In a subgroup analysis of the DOREMI trial [20], patients receiving BBs 24 h prior to undergoing CS had fewer episodes of cardiac arrest and lower mortality in the early resuscitative period of CS, compared with those who did not receive BB treatment (despite this, these benefits were not maintained throughout the hospitalization, and there was no difference in mortality at discharge).

From a pharmacological point of view, the concomitant use of BBs and inotropic support could appear contradictory. However, in the setting of AHF patients requiring inotropic support, BBs have been shown to reduce the rate of vasopressor/inotrope-induced ventricular arrhythmias and the rates of premature ventricular contractions [21]. Moreover, Böhm and colleagues showed in a multicenter, randomized, double-blinded study that in patients with AHF who required inotropes, BBs at admission and discharge resulted in lower mortality at 31 days [22]. Additionally, in a retrospective single-center study, Delmas and al. demonstrated a protective association between BBs at admission and long-term mortality in patients with CS [23]. Finally, medically guided therapy for HF (including, of course, BBs) in CS survivors significantly reduced one-year mortality (in press).

In our study, we compared the patients taking or not taking BB at 24 h after the CS event. The two groups showed overlapping characteristics regarding major comorbidities, although patients in the BB group had more hypertension and diabetes, which are recognized strong predictors of mortality. Although the study could not answer why patients were previously taking BBs (e.g., heart failure, arrhythmias, myocardial infarction, etc.), no differences were found in terms of CS triggers between the two groups. In fact, the two groups of patients did not differ in terms of history of heart disease and CS triggers. However, it is noteworthy that patients without BBs at 24 h showed generally more severe clinical features than those taking BBs (worse LVEF, greater use of inotropes/vasopressors, invasive ventilation, and aMCS). BB use at 24 h was not associated with short- or long-term mortality. We did not show significant differences between groups in 1-month and 1-year all-cause mortality after adjustment but only positive trends (aHR = 0.61, 95% CI 0.34–1.1, *p* = 0.10 and aHR = 0.74, 95% CI 0.5–1.08, *p* = 0.12, respectively).

In this observational study, BB prescription at 24 h rather correlated with a modest shock grade, which is in line with the negation of the BB effect on mortality after adjustment for shock severity factors. Importantly, a protective role of BB continuation in the acute phase of CS is suggested here by the lower mortality at 1 month even after adjustment for CS severity factors (aHR 0.43, 95% CI 0.2–0.92, *p* = 0.03), in line with previous literature [20,23,24]. The explanations for this protective effect of BBs have not yet been fully elucidated. Underlying it could be an adrenergic rebound effect in response to abrupt BB discontinuation [11,25], damage to cardiac contractility [26], or even increased sensitivity to endogenous (or exogenous) catecholamines [27,28]. Such an analysis is beyond the scope of our study, and randomized clinical trials remain mandatory to address this issue. Nevertheless, BB continuation is associated with reduced mortality, supporting their use in patients with less severe disease.

Consistently, the early introduction (within 24 h) of BB therapy after CS was not associated with changes in both 1-month (aHR = 0.96, 95% CI 0.38–2.4, *p* = 0.93) and 1-year all-cause mortality (aHR = 0.67, 95% CI 0.33–1.39, *p* = 0.28). However, looking at the mortality curves, it seems that the introduction of BBs before discharge, rather than early introduction within 24 h, may have a strong positive impact on prognosis. This finding is in line with the literature, in which the intrahospital use of BBs could help to achieve the cardioprotective effect of this class of drugs earlier and titrate optimal medical therapy with a prognostic impact on these patients at high risk of death.

### Strengths and Limitations

Our work prospectively included 772 consecutive CS patients (the larger European cohort) from a broad spectrum of etiologies. In addition, we used a contemporary and pragmatic definition of CS that considerably strengthens our results. However, the more current SCAI SHOCK stage classification [29] was not used for group classification, as this score was not available at the time of the study.

We acknowledge the following limitations. First, this was an observational study with its associated, well-known bias, but it reflects real-life practice in different settings based on many investigating centers and units. Real-world registries are an important source for exploring optimal medical treatments; nevertheless, the results from our analysis should be considered only as hypothesis-generating. Consistently, inclusion criteria were not too stringent to give a real-life picture of patients considered as presenting CS. We cannot exclude a bias with the recruitment of patients on the edge of CS as classically defined. This is not a real limitation regarding this work, as the discontinuation of patients considered in the continuum of the wide spectrum of patients admitted for AHF leading to CS remains a real concern in clinical practice. Second, this prospective analysis of FRENSHOCK lacks information on the achieved up-titrated drug doses and on guideline-directed medical therapy changes during follow-up. Third, we are unable to distinguish the type of BB used (e.g., bisoprolol, metoprolol, and propranolol), so the results are about a class effect and not about the specific drug. Fourth, the duration of BB therapy before hospitalization is not known, and the duration of BB therapy beyond the time of discharge was not collected. It is likely that in the group of patients who discontinued BBs, there are patients to whom BBs were introduced at hospital discharge or later during follow-up, which creates a confounding bias. In addition, we do not know the duration of pre-existing treatments, so our conclusions should be cautiously interpreted. Finally, although we attempted to assess the relationship between CS and post-hospital outcomes by adjusting for a broad range of clinical factors and treatments, the possibility of confounding by unmeasured covariates remains.

## 4. Materials and Methods

### 4.1. Patient Population and Data Collection

This was an ancillary analysis of the FRENSCHOCK registry, which is a prospective, observational, and multicenter registry (NCT02703038) conducted over a 6-month period in France between April and October 2016, including patients admitted for CS in the intensive care unit (ICU) and CCU at 49 centers in France, coming from all types of institutions (from primary to tertiary centers, both university and non-university, and public and private hospitals) [30,31].

All patients (*n* = 772) presenting with CS were included if they met at least one criterion of each of the following: (1) low cardiac output: low systolic blood pressure (SBP) < 90 mmHg, the need for maintenance with vasopressors/inotropes, and/or a low cardiac index < 2.2 L/min/m^2^; (2) left and/or right heart filling pressure elevation, defined using clinical signs, radiology, blood tests, echocardiography, or signs of invasive hemodynamic overload; and (3) signs of organ malperfusion, which could be clinical (oliguria, confusion, pale and/or cold extremities, and mottled skin) and/or biological (lactate > 2 mmol/L, metabolic acidosis, renal failure, and liver insufficiency). Patients could be included regardless of CS etiology and whether CS was present at admission or developed during their in-hospital course. Exclusion criteria were refusal to participate, shock from a non-cardiac origin, and post-cardiotomy CS.

For each patient, investigators had to specify one to three CS triggers among the following: ischemic (type 1 or 2 acute myocardial infarction (AMI)), mechanical complications (valvular injury or ventricular septal defect), ventricular and supraventricular arrhythmia, severe bradycardia, iatrogenesis (medication-induced), infections, and non-observance of previous medication.

Past medical history, ongoing treatments, and clinical, biological, and echocardiographic data were collected at admission and at 24 h, as previously described [30,31]. In-hospital CS management (especially inotropes/vasopressors, mechanical ventilation, renal replacement therapy, and acute mechanical circulatory support (aMCS)) was also reported.

### 4.2. Study Endpoints and Follow-Up

The primary and secondary endpoints were all-cause mortality at 1 month and 1 year, respectively. The follow-up was completed on the last medical interview date, the last examination date, or the date when the outcome event occurred. At 1 month and at 1 year, follow-up was performed using the following sequential procedures: first, consult the registry office of the patient’s birthplace for death certificates; next, contact the patient’s general practitioner and/or cardiologist; and finally, contact the patient or their direct relatives.

### 4.3. Statistical Analysis

Continuous variables were presented as mean and standard deviation (SD) or medians and interquartile ranges (IQRs) when appropriate. Categorical variables were summarized in terms of counts and percentages. Comparisons of continuous variables between groups were performed with the Mann–Whitney non-parametric test. Categorical variables were compared with the chi-square test or Fisher’s exact test when suitable. Paired data were analyzed with the Wilcoxon signed-rank test.

The main analysis was a comparison between CS patients with and without BB treatment after 24 h of management. To investigate other common scenarios, further analyses were conducted regarding non-discontinuation and early introduction of BBs at 24 h.

To determine independent predictors for each primary and secondary outcome, multivariate stepwise logistic regression analysis was performed. The covariates included in the model consisted of all baseline characteristics (age, sex ratio, body mass index, cardiovascular risk factors, comorbidities, New York Heart Association (NYHA) functional class, history of previous heart disease, medications, initial cardiac arrest, and sinus rhythm), as well as treatment modalities (diuretics, norepinephrine, epinephrine, dobutamine, renal replacement therapy, aMCS, and invasive and non-invasive ventilation), CS triggers (ischemic, mechanical complication, ventricular and supraventricular arrhythmia, infections, non-observance, and iatrogenesis), and markers of CS severity (LVEF ≤ 30%, lactates ≥ 4 mmol/L, and estimated glomerular filtration rate (eGFR) ≤ 30 mL/min). First, the association between these characteristics and each outcome of interest was assessed using univariable logistic regression analyses. Thereafter, all significant independent predictors were integrated into multivariable analyses for each outcome and then reduced to only significant characteristics (*p* ≤ 0.05). Finally, these significant characteristics were incorporated in multivariable models as fixed covariates for each adjusted outcome analysis. Significant risk factors were reported with their respective odds ratios (ORs) and 95% confidence intervals (CIs). The variance inflation factor was used to rule out multicollinearity among the variables. Primary and secondary outcomes were assessed using Kaplan–Meier time-to-event analysis, and Cox proportional hazards models were used to determine the adjusted hazard ratio (aHR), 95% CI, and *p* values.

Analyses were performed using R software (version 4.1.2 (2021-11-01)). A *p* value < 0.05 was considered statistically significant.

## 5. Conclusions

In patients with CS from multiple etiologies, BBs were not associated with an increase in early all-cause mortality. By contrast, the discontinuation of BB therapy was associated with an increase in 1-month mortality and an increasing trend in 1-year all-cause mortality. Considering all of these data, the impact of BBs appears promising although challenging in CS patients. Some limitations (details on the drugs, durations, doses, etc.), however, prevent firm conclusions. These results support the proposal for a randomized clinical trial that could definitively provide answers on the management of betablockers in patients with cardiogenic shock.

## Figures and Tables

**Table 1:** Baseline clinical and echocardiographic characteristics at admission according to treatment with betablockers at 24 h (BB vs. non-BB).

	Overall Population (*n* = 693)	BB Group (*n* = 95)	Non-BB Group (*n* = 598)	*p* Value
Age, mean ± SD, years	66 ± 14.6	68.3 ± 15.1	65.7 ± 14.5	0.07
Male, *n* (%)	495 (71.4)	53 (55.8)	442 (73.9)	<0.01
Body mass index, mean ± SD, kg/m²	25.7 ± 5.5	26.5 ± 6.5	25.6 ± 5.3	0.44
Risk factors, *n* (%)				
Diabetes mellitus	195 (28.2)	36 (37.9)	159 (26.6)	0.03
Hypertension	329 (47.5)	57 (60)	272 (45.5)	0.01
Dyslipidemia	254 (36.7)	40 (42.1)	214 (35.8)	0.25
Current smoker	183 (27.4)	28 (30.1)	155 (27)	0.53
Medical history, *n* (%)				
Peripheral artery disease	84 (12.1)	13 (13.7)	71 (11.9)	0.61
Chronic kidney disease	151 (21.8)	21 (22.1)	130 (21.8)	1
COPD	44 (6.4)	6 (6.3)	38 (6.4)	1
ICD	111 (16)	18 (18.9)	93 (15.6)	0.45
Active cancer	47 (6.8)	3 (3.2)	44 (7.4)	0.18
Stroke	54 (7.8)	10 (10.5)	44 (7.4)	0.3
NYHA functional status, *n* (%)				
≥3	265 (39.2)	34 (37)	231 (39.6)	0.76
History of cardiac disease, *n* (%)				
All causes	392 (56.6)	57 (60)	335 (56)	0.5
Ischemic	205 (29.6)	35 (36.8)	170 (28.4)	0.12
Hypertrophic	11 (1.6)	2 (2.1)	9 (1.5)	0.65
Toxic	29 (4.2)	4 (4.2)	25 (4.2)	1
Dilated	72 (10.4)	9 (9.5)	63 (10.5)	0.86
Valvular	58 (8.4)	5 (5.3)	53 (8.9)	0.32
Hypertensive	22 (3.2)	2 (2.1)	20 (3.3)	0.76
Previous medications, *n* (%)				
Aspirin	255 (36.8)	33 (34.7)	222 (37.1)	0.73
P2Y12 inhibitors	120 (17.3)	12 (12.6)	108 (18.1)	0.24
Oral anticoagulant (VKA or DOAC)	201 (29)	39 (41.1)	162 (27.1)	< 0.01
ACEi, ARB or ARNi	271 (40.2)	46 (48.9)	225 (38.8)	0.07
Statins	256 (36.9)	37 (38.9)	219 (36.6)	0.73
Loop diuretics	340 (49.1)	56 (58.9)	284 (47.5)	0.046
Aldosterone antagonist	100 (14.4)	9 (9.5)	91 (15.2)	0.16
Cardiogenic shock triggers, *n* (%)				
Ischemic	254 (36.7)	42 (44.2)	212 (35.5)	0.11
Non-ischemic	365 (52.7)	44 (46.3)	321 (53.7)	
Supraventricular tachycardia	96 (13.9)	13 (13.7)	83 (13.9)	1
Infectious disease	80 (11.5)	8 (8.4)	72 (12)	0.39
Ventricular arrhythmia	86 (12.4)	15 (15.8)	71 (11.9)	0.31
Iatrogenesis	41 (5.9)	3 (3.2)	38 (6.4)	0.35
Non-observance	27 (3.9)	3 (3.2)	24 (4)	1
Mechanical complications	21 (3)	2 (2.1)	19 (3.2)	0.76
Conduction disorder	14 (2)	0 (0)	14 (2.3)	0.24
Clinical presentation at admission				
Heart rate, mean ± SD, bpm	95.4 ± 29.7	100.9 ± 35.9	94.6 ± 28.6	0.33
SBP, mean ± SD, mmHg	102 ± 25.1	108.2 ± 20.9	101.1 ± 25.6	<0.01
Sinus rhythm, *n* (%)	355 (51.4)	35 (36.8)	320 (53.8)	<0.01
Cardiac arrest, *n* (%)	66 (9.5)	11 (11.6)	55 (9.2)	0.45
Blood tests at admission, median (IQR)				
Sodium, mmol/L	135 (132–139)	137 (133.5–140)	135 (131–139)	0.03
Creatinin, μmol/L	132.5 (94–188)	116 (83.5–152)	134 (97–194)	0.01
Bilirubin, mg/L	16 (9.5–28)	18 (10–30)	16 (9.1–28)	0.51
Haemoglobin, g/dL	12.7 (11–14)	12 (10.6–13.6)	13 (11–14)	0.049
Arterial blood lactates, mmol/L	3.0 (2.0–4.2)	2.2 (1.9–3.0)	3.0 (2.0–5.0)	<0.01
ASAT, UI/L	87 (38.3–292.5)	51 (36–180)	91 (39–301)	0.08
Nt-proBNP, pg/mL	10,293.5 (4442.5–26,322.5)	5922 (4846–13,701)	10,626 (4389–27,884)	0.23
Baseline echocardiography				
LVEF, mean ± SD, %	26.7 ± 13.3	29.9 ± 13	26.1 ± 13.3	<0.01
TAPSE, median (IQR), mm	13 (10–17)	13 (10–16)	13 (10–17)	0.85
PSVtdi, median (IQR), cm/s	8 (6–10)	8 (7–10.3)	8 (6–10)	0.77
Severe mitral regurgitation, *n* (%)	96 (14.6)	8 (8.6)	88 (15.5)	0.08
Severe aortic stenosis, *n* (%)	31 (4.5)	4 (4.3)	27 (4.6)	1
Severe aortic regurgitation, *n* (%)	9 (1.3)	1 (1.1)	8 (1.4)	1

The total number of participants may vary for certain variables due to missing data. ACE = angiotensin-converting enzyme, ARB = angiotensin receptor blocker, ARNi = angiotensin receptor II blocker-neprilysin inhibitor, ASAT = aspartate aminotransferase, BB = betablocker, COPD = chronic obstructive pulmonary disease, DOAC = direct oral anticoagulant ICD = implantable cardioverter–defibrillator, IQR = interquartile range, LVEF = left ventricular ejection fraction, NYHA = New York Heart Association, Nt-proBNP = N-terminal-pro hormone brain natriuretic peptide, PSVtdi = peak systolic velocity tissue Doppler imaging, SBP = systolic blood pressure, SD = standard deviation, TAPSE = tricuspid annular plane systolic excursion, VKA = vitamin K antagonists.

**Table 2:** In-hospital management according to treatment with betablockers at 24 h (BB vs. non-BB).

	Overall Population (*n* = 693)	BB Group (*n* = 95)	Non-BB Group (*n* = 598)	*p* Value
Medications used, *n* (%)				
Dobutamine	566 (81.8)	59 (62.1)	507 (84.9)	<0.01
Norepinephrine	361 (52.2)	25 (26.3)	336 (56.3)	<0.01
Epinephrine	87 (12.6)	10 (10.5)	77 (12.9)	0.62
Levosimendan	50 (7.2)	4 (4.2)	46 (7.7)	0.29
Loop diuretics	467 (67.4)	73 (76.8)	394 (65.9)	0.03
Respiratory support, *n* (%)				
Non-invasive	184 (26.6)	43 (45.7)	141 (23.6)	<0.01
Invasive	249 (36)	15 (15.8)	234 (39.2)	<0.01
Short-term mechanical circulatory support, *n* (%)	121 (17.5)	9 (9.5)	112 (18.8)	0.03
Renal replacement therapy, *n* (%)	107 (15.4)	7 (7.4)	100 (16.7)	0.02
Any PCI, *n* (%)	206 (70.8)	27 (75)	179 (0.2)	0.7

The total number of participants may vary for certain variables due to missing data. BB = betablocker, PCI = percutaneous coronary intervention.

## Data Availability

Data are contained within the article and Supplementary Materials.

## Supplementary Materials

The following supporting information can be downloaded at: https://www.mdpi.com/article/10.3390/ph16121740/s1.
