# Supplementary material for: Beta Blockers Improve Prognosis When Used Early in Patients with Cardiogenic Shock: An Analysis of the FRENSHOCK Multicenter Prospective Registry
Source: Pharmaceuticals (Basel). 2023 Dec 18;16(12):1740. doi: 10.3390/ph16121740 (PMC10747751; doi:10.3390/ph16121740)
Supplement: Supplementary file 1 [file pharmaceuticals-16-01740-s001.zip › pharmaceuticals-2728937-supplementary.pdf]

Table S1. Baseline clinical and echocardiographic characteristics at admission according to discontinuation of betablockers within the first 24 h *.

	Overall Population (*n* = 286)	BB Non-Discontinued (*n* = 63)	BB Discontinued (*n* = 223)	*p* Value
Age, mean ± SD, years	68.7 ± 13.6	69.4 ± 15.2	68.5 ± 13.1	0.34
Male, n (%)	187 (65.4)	28 (44.4)	159 (71.3)	<0.01
Body mass index, mean ± SD, kg/m²	26 ± 5.7	26.3 ± 6.4	26 ± 5.5	0.98
Risk factors, n (%)				
Diabetes mellitus	101 (35.3)	23 (36.5)	78 (35)	0.88
Hypertension	169 (59)	39 (61.9)	130 (58.3)	0.66
Dyslipidemia	134 (46.9)	27 (42.9)	107 (48)	0.48
Current smoker	61 (22)	17 (27.9)	44 (20.4)	0.22
Medical history, n (%)				
Peripheral artery disease	45 (15.7)	8 (12.7)	37 (16.6)	0.56
Chronic kidney disease	88 (30.8)	17 (27)	71 (31.8)	0.54
COPD	22 (7.7)	5 (8)	17 (7.6)	1
ICD	68 (23.8)	14 (22.2)	54 (24.2)	0.87
Active cancer	18 (6.3)	2 (3.2)	16 (7.2)	0.38
Stroke	29 (10.1)	8 (12.7)	21 (9.4)	0.48
NYHA functional status, n (%)				
≥ 3	119 (42.7)	25 (40.3)	94 (43.3)	0.77
History of cardiac disease, n (%)				
All causes	223 (78)	45 (71.4)	178 (80)	0.17
Ischemic	125 (43.7)	26 (41.3)	99 (44.4)	0.67
Hypertrophic	8 (2.8)	2 (3.2)	6 (2.7)	1
Toxic	12 (4.2)	3 (4.8)	9 (4)	0.73
Dilated	44 (15.4)	8 (12.7)	36 (16.1)	0.56
Valvular	25 (8.7)	5 (8)	20 (9)	1
Hypertensive	11 (3.8)	1 (1.6)	10 (4.5)	0.47
Previous medications, n (%)				
Aspirin	129 (45.1)	26 (41.3)	103 (46.2)	0.57
P2Y12 inhibitors	54 (18.9)	7 (11.1)	47 (21.1)	0.1
Oral anticoagulant (VKA or DOAC)	122 (42.7)	30 (47.6)	92 (41.3)	0.39
ACEi, ARB or ARNi	168 (58.9)	33 (53.2)	135 (60.5)	0.31
Statins	153 (53.5)	28 (44.4)	125 (56.1)	0.12
Loop diuretics	194 (67.8)	44 (69.8)	150 (67.3)	0.76
Aldosterone antagonist	67 (23.4)	8 (12.7)	59 (26.5)	0.03
Cardiogenic Shock triggers, n (%)				

Ischemic	88 (30.8)	25 (39.7)	63 (28.3)	0.1
Non-ischemic	164 (57.3)	28 (44.4)	136 (61)	
Supraventricular tachycardia	48 (16.8)	9 (14.3)	39 (17.5)	0.7
Infectious disease	31 (10.8)	6 (9.5)	25 (11.2)	0.82
Ventricular arrhythmia	34 (11.9)	9 (14.3)	25 (11.2)	0.51
Iatrogenesis	12 (4.2)	2 (3.2)	10 (4.5)	1
Non-observance	22 (7.7)	1 (1.6)	21 (9.4)	0.06
Mechanical complications	7 (2.4)	1 (1.6)	6 (2.7)	1
Conduction disorder	10 (3.5)	0 (0)	10 (4.5)	0.12
Clinical presentation at admission				
Heart rate, mean ± SD, bpm	91.2 ± 29.9	97 ± 34.6	89.5 ± 28.2	0.27
SBP, mean ± SD, mmHg	100.8 ± 25.1	106.7 ± 20.7	99.1 ± 26	<0.01
Sinus rhythm, n (%)	124 (43.4)	22 (34.9)	102 (45.7)	0.15
Cardiac arrest, n (%)	20 (7)	6 (9.5)	14 (6.3)	0.4
Blood tests at admission, median (IQR)				
Sodium, mmol/L	135 (131.3–138)	136 (134–139.5)	135 (131–137)	<0.01
Creatinin, µmol/L	141 (97–202.8)	118 (83.5–176)	143 (106–205.5)	0.02
Bilirubin, mg/L	18 (10–29)	18 (10.3–30.8)	17 (10–28)	0.77
Haemoglobin, g/dL	12 (10.6–14)	12 (10.5–13.8)	12.1 (10.7–14)	0.39
Arterial blood lactates, mmol/L	2.7 (2–4)	2.3 (1.9–3)	3 (2–4)	0.02
ASAT, UI/L	71.5 (35–223)	49 (37–93)	78 (34–281.5)	0.15
Nt-proBNP, pg/mL	12,652 (5097–28,554)	6787 (5228.5–18,347.5)	12,710.5 (5073.3–29,692)	0.5
Baseline echocardiography				
LVEF, mean ± SD, %	27.2 ± 13.5	30.2 ± 13.4	26.3 ± 13.4	0.03
TAPSE, median (IQR), mm	13 (10–16)	13 (10–16)	14 (10–16)	0.96
PSVtdi, median (IQR), cm/s	7.5 (6–10)	7 (7–9.5)	8 (6–10)	0.71
Severe mitral regurgitation, n (%)	47 (17.1)	6 (9.7)	41 (19.2)	0.09
Severe aortic stenosis, n (%)	12 (4.3)	4 (6.5)	8 (3.6)	0.31
Severe aortic regurgitation, n (%)	2 (0.7)	1 (1.6)	1 (0.5)	0.4

* The total number of participants may vary for certain variables due to missing data. * ACE = angiotensin-converting enzyme, ARB = angiotensin receptor blocker, ARNi = angiotensin receptor II blocker-neprilysin inhibitor, ASAT = aspartate aminotransferase, BB = betablocker, COPD = chronic obstructive pulmonary disease, DOAC = direct oral anticoagulant ICD = implantable cardioverter–defibrillator, IQR = interquartile range, LVEF = left ventricular ejection fraction, NYHA = New York Heart Association, Nt-proBNP = N-terminal-pro hormone brain natriuretic peptide, PSVtdi = peak systolic velocity tissue Doppler imaging, SBP = systolic blood pressure, SD = standard deviation, TAPSE = tricuspid annular plane systolic excursion, VKA = vitamin K antagonists.

Table S2. In-hospital management according to discontinuation of betablockers within the first 24 h *.

	Overall Population (*n* = 286)	BB Non-Discontinued (*n* = 63)	BB Discontinued (*n* = 223)	*p* Value
Medications used, n (%)				
Dobutamine	226 (79.3)	37 (58.7)	189 (85.1)	<0.01
Norepinephrine	128 (44.9)	14 (22.2)	114 (51.4)	<0.01
Epinephrine	32 (11.2)	5 (7.9)	27 (12.1)	0.5
Levosimendan	22 (7.7)	3 (4.8)	19 (8.6)	0.43
Loop diuretics	203 (71)	47 (74.6)	156 (70)	0.53
Respiratory support, n (%)				
Non-invasive	74 (26)	28 (44.4)	46 (20.7)	<0.01
Invasive	78 (27.4)	8 (12.7)	70 (31.5)	<0.01
Short-term mechanical circulatory support, n (%)	32 (11.3)	3 (4.8)	29 (13.1)	0.07
Renal replacement therapy, n (%)	39 (13.6)	5 (7.9)	34 (15.2)	0.15
Any PCI, n (%)	64 (65.3)	15 (83.3)	49 (61.3)	0.1

* The total number of participants may vary for certain variables due to missing data. * *BB = betablocker, PCI = percutaneous coronary intervention.*

Table S3. Baseline clinical and echocardiographic characteristics at admission according to early introduction of betablockers (early BB vs. non-early BB).

	Overall Population (*n* = 407)	Early BB (*n* = 32)	Non-Early BB (*n* = 375)	p Value
Age, mean ± SD, years	64.1 ± 15	66.2 ± 14.7	64 ± 15.1	0.53
Male, n (%)	308 (75.7)	25 (78.1)	283 (75.5)	0.83
Body mass index, mean ± SD, kg/m²	25.5 ± 5.3	26.9 ± 6.8	25.4 ± 5.1	0.31
Risk factors, n (%)				
Diabetes mellitus	94 (23.2)	13 (40.6)	81 (21.7)	0.03
Hypertension	160 (39.3)	18 (56.3)	142 (37.9)	0.06
Dyslipidemia	120 (29.5)	13 (40.6)	107 (28.5)	0.16
Current smoker	122 (31.2)	11 (34.4)	111 (30.9)	0.69
Medical history, n (%)				
Peripheral artery disease	39 (9.6)	5 (15.6)	34 (9.1)	0.22
Chronic kidney disease	63 (15.5)	4 (12.5)	59 (15.8)	0.8
COPD	22 (5.4)	1 (3.1)	21 (5.6)	1
ICD	43 (10.6)	4 (12.5)	39 (10.4)	0.76
Active cancer	29 (7.1)	1 (3.1)	28 (7.5)	0.72
Stroke	25 (6.2)	2 (6.3)	23 (6.1)	1
NYHA functional status, n (%)				
≥3	146 (36.8)	9 (30)	137 (37.3)	0.55

History of cardiac disease, n (%)				
All causes	169 (41.5)	12 (37.5)	157 (41.9)	0.71
Ischemic	80 (19.7)	9 (28.1)	71 (18.9)	0.37
Hypertrophic	3 (0.7)	0 (0)	3 (0.8)	1
Toxic	17 (4.2)	1 (3.1)	16 (42.7)	1
Dilated	28 (6.9)	1 (3.1)	27 (7.2)	0.71
Valvular	33 (8.1)	0 (0)	33 (8.8)	0.09
Hypertensive	11 (2.7)	1 (3.1)	10 (2.7)	0.6
Previous medications, n (%)				
Aspirin	126 (31)	7 (21.9)	119 (31.7)	0.32
P2Y12 inhibitors	66 (16.2)	5 (15.6)	61 (16.3)	1
Oral anticoagulant (VKA or DOAC)	79 (19.4)	9 (28.1)	70 (18.7)	0.24
ACEi, ARB or ARNi	102 (26.2)	13 (40.6)	89 (24.9)	0.06
Statins	103 (25.3)	9 (28.1)	94 (25.1)	0.68
Loop diuretics	146 (35.9)	12 (37.5)	134 (35.7)	0.85
Aldosterone antagonist	33 (8.1)	1 (3.1)	32 (8.5)	0.5
Cardiogenic shock triggers, n (%)				
Ischemic	166 (40.8)	17 (53.1)	149 (39.7)	0.19
Non-ischemic	201 (49.4)	16 (50)	185 (49.3)	
Supraventricular tachycardia	48 (11.8)	4 (12.5)	44 (11.7)	0.78
Infectious disease	49 (12)	2 (6.3)	47 (12.5)	0.4
Ventricular arrhythmia	52 (12.8)	6 (18.8)	46 (12.3)	0.28
Iatrogenesis	19 (4.7)	2 (6.3)	17 (4.5)	0.65
Non-observance	15 (3.7)	1 (3.1)	14 (3.7)	1
Mechanical complications	14 (3.4)	1 (3.1)	13 (3.5)	1
Conduction disorder	4 (1)	0 (0)	4 (1.1)	1
Clinical presentation at admission				
Heart rate, mean ± SD, bpm	98.4 ± 29.3	108.7 ± 37.7	97.5 ± 28.4	0.21
SBP, mean ± SD, mmHg	103 ± 25.1	111.2 ± 21.2	102.2 ± 25.3	<0.01
Sinus rhythm, n (%)	231 (57.2)	13 (40.6)	218 (58.6)	0.06
Cardiac arrest, n (%)	46 (11.3)	5 (15.6)	41 (10.9)	0.39
Blood tests at admission, median (IQR)				
Sodium, mmol/L	136 (132–139)	137 (132.8–140)	136 (132–139)	0.4
Creatinin, µmol/L	124 (93–172.8)	113.5 (83.5–134.3)	125 (93–179.5)	0.09
Bilirubin, mg/L	15 (9–28)	13.5 (10–26.3)	15 (9–28)	0.96
Haemoglobin, g/dL	13 (11–14.2)	12.1 (11.8–13)	13 (11–14.4)	0.22
Arterial blood lactates, mmol/L	3 (2–5)	2 (1.7–2.9)	3 (2–5)	0.047
ASAT, UI/L	102 (42–360.3)	82.5 (34.5–363)	102.5 (42.8–360.3)	0.76

Nt-proBNP, pg/mL	8388 (3357–21,306)	4776.5 (1249.5–9155.5)	8499 (3419–22,149)	0.12
Baseline echocardiography				
LVEF, mean ± SD, %	26.3 ± 13.1	29.4 ± 12.2	26 ± 13.2	0.08
TAPSE, median (IQR), mm	13 (10–17)	12.5 (10.5–14)	13 (10–17)	0.67
PSVtdi, median (IQR), cm/s	8 (6–10)	8 (8–10)	8.5 (6–10)	0.71
Severe mitral regurgitation, n (%)	49 (12.8)	2 (6.5)	47 (13.3)	0.4
Severe aortic stenosis, n (%)	19 (4.8)	0 (0)	19 (5.2)	0.38
Severe aortic regurgitation, n (%)	7 (1.8)	0 (0)	7 (1.9)	1

The total number of participants may vary for certain variables due to missing data. ACE = angiotensin-converting enzyme, ARB = angiotensin receptor blockers, ARNi = angiotensin receptor II blocker-neprilysin inhibitor, ASAT = aspartate aminotransferase, BB = betablocker, COPD = chronic obstructive pulmonary disease, DOAC = direct oral anticoagulant ICD = implantable cardioverter–defibrillator, IQR = interquartile range, LVEF = left ventricular ejection fraction, NYHA = New York Heart Association, Nt-proBNP = N-terminal-pro hormone brain natriuretic peptide, PSVtdi = peak systolic velocity tissue Doppler imaging, SBP = systolic blood pressure, SD = standard deviation, TAPSE = tricuspid annular plane systolic excursion, VKA = vitamin K antagonists.

Table S4. In-hospital management according to early introduction of betablockers (early BB vs. non-early BB).

	**Overall Population (*n* = 407)**	**Early BB (*n* = 32)**	**Non-Early BB (*n* = 375)**	***p* Value**
Medications used, n (%)				
Dobutamine	340 (83.5)	22 (31.3)	318 (84.8)	0.03
Norepinephrine	233 (57.2)	11 (34.4)	222 (59.2)	<0.01
Epinephrine	55 (13.5)	5 (15.6)	50 (13.3)	0.79
Levosimendan	28 (6.9)	1 (3.1)	27 (7.2)	0.71
Loop diuretics	264 (64.9)	26 (81.3)	238 (63.5)	0.053
Respiratory support, n (%)				
Non-invasive	110 (27)	15 (46.9)	95 (25.3)	0.01
Invasive	171 (42)	7 (21.9)	164 (43.7)	0.02
Short-term mechanical circulatory support, n (%)	82 (20.1)	6 (18.8)	76 (20.3)	1
Renal replacement therapy, n (%)	68 (16.7)	2 (6.3)	66 (17.6)	0.14
Any PCI, n (%)	143 (74.1)	12 (66.7)	131 (74.9)	0.57

The total number of participants may vary for certain variables due to missing data. BB = betablocker, PCI = percutaneous coronary intervention.
